# Recycling of Chinese herb residues by endophytic and probiotic fungus *Aspergillus cristatus* CB10002 for the production of medicinal valuable anthraquinones

**DOI:** 10.1186/s12934-019-1150-9

**Published:** 2019-06-04

**Authors:** Wenping Kong, Chengshuang Huang, Jie Shi, Yu Li, Xinxin Jiang, Quwen Duan, Yong Huang, Yanwen Duan, Xiangcheng Zhu

**Affiliations:** 1 Xiangya International Academy of Translational Medicine, Central South University, Tongzipo Road, #172, Yuelu District, Changsha, 410013 Hunan China; 2 Hayaocihang Pharmaceutical Co. Ltd, Changsha, 410205 Hunan China; 3 Hunan Engineering Research Center of Combinatorial Biosynthesis and Natural Product Drug Discovery, Changsha, 410205 Hunan China; 4 National Engineering Research Center of Combinatorial Biosynthesis for Drug Discovery, Changsha, 410013 Hunan China

**Keywords:** Herb residues, Microbial recycling, *Aspergillus cristatus*, Solid state fermentation, Medicinal valuable anthraquinones

## Abstract

**Background:**

The global prevalence of traditional Chinese medicine stimulates the prosperous development of herb medicines, but the annual generation of massive herb residues becomes big issues about environmental pollution and waste of resources. Microbes play important roles in the circulation of substances in nature, and endophytes represent an underexplored microbial resource possessing the unique symbiotic relationship with plants, not only for discovery of secondary metabolites, but also for potential green recycling of herb residues.

**Results:**

The recycling capacities of several endophytic strains were respectively evaluated via solid state fermentation with herb residues of commercial Huazhenghuisheng oral-liquid (HOL). Among them, *Aspergillus cristatus* CB10002, a probiotic fungus isolated from Chinese Fu-brick tea, was competent to recycle HOL residues for the production of medicinal valuable anthraquinones, in which four of them, especially citreorosein with significant anti-obesity activity, were first discovered in *A. cristatus*. Subsequent quantitative analysis showed that about 2.0 mg/g citreorosein and 7.5 mg/g total anthraquinones could be obtained after 35-day fermentation, which was very competitive and economically beneficial. Further nutritional comparisons also revealed that the recycling process indeed ameliorated the nutrients of HOL residues, and thus proposed a possibility to directly dispose the final leftovers as a compost organic fertilizer.

**Conclusions:**

The endophytic and probiotic fungus *A. cristatus* CB10002 isolated from Chinese Fu-brick tea was screened out to effectively reutilize HOL residues for the production of nine medicinal valuable anthraquinones, whose biosynthesis may be regulated by the induction of HOL residues. The competitive yields of these anthraquinones, as well as the certain composting properties of final leftovers, have made the microbial recycling of HOL residues economically beneficial. Our work demonstrated a promising applied potential of *A. cristatus* in reutilization of herb residues, and provided a practical strategy for sustainable and value-added microbial recycling of herb residues.

**Electronic supplementary material:**

The online version of this article (10.1186/s12934-019-1150-9) contains supplementary material, which is available to authorized users.

## Background

Traditional Chinese medicine (TCM) is one of the best heritages and the essential part of healthcare system in China with a very long history [1]. Since TCM has presented good efficacy in the treatment of many chronic diseases like diabetes and cardiovascular diseases [2], it has also been extensively applied in other Asian countries, and considered as a complementary or alternative medical treatment worldwide [3, 4]. According to the World Health Organization (WHO), TCM is applied for health care by about 80% of populations, and it will soon be recognized in the global medical compendium of WHO [3]. The global prevalence of TCM has promoted rapid growth of Chinese herbal medicine (CHM) in China, where CHM has already represented around 40% of the pharmaceutical market with annual sales of 21 billion dollars at 2012 [1]. However, the prosperous development of CHM industries have also generated more than 30 million tons herb residues every year, and most of them are solid wastes derived after the extraction of water or ethanol [5]. In spite of remaining nutrients and/or bioactive substances, the massive herb residues have been directly disposed through stacking, landfill or burning [6, 7], which causes serious environmental pollution and waste of resources. Although some herb residues could be reused as feed additives, raw material for papermaking and cultivation medium of edible fungi [5], special requirements about the types of herb residues have limited the extension of such methods. Many efforts have been devoted to explore general recycling strategies for herb residues, such as pyrolysis [8] or gasification [7] to obtain fuel gas, or conversion into biochar [9], but these approaches ignored the potential nutritional and medicinal ingredients of herb residues and are normally high energy consuming, which are not beneficial for green and practical applications.

Microorganisms possess diversified enzymes and play important roles in the circulation and conversion of substances in nature [10]. The microbial conversion of CHM has been adopted since ancient times to improve medical effects or reduce toxicities [11], which also inspired bioconversions for recycling of herb residues, with more concerns about their possible medicinal or nutritional values. The fermentation treatment with *Aspergillus oryzae* could improve the antioxidant and antibacterial activities of four common herb residues [12]; while the fermentation supernatant of herb residues (from Jianweixiaoshi tablet) cultivating with several probiotics also showed potential inhibition activities towards *Helicobacter pylori* infection or antibiotic-associated diarrhea [5]. Recent composting studies have indicated that the active ingredients within herb residues seemed not specifically affect the microbial diversity, but could induce changes in the antagonistic and parasitic abilities of microbes, and resulted in the enhanced anti pathogenic property of residue compost [6]. Although the specific molecules or mechanisms were not clarified, these preliminary studies demonstrated the feasibility and great potential for microbial recycling of herb residues. Endophytes are bacteria or fungi ubiquitously inhabiting in different plants, and the unique mutualism between them has evolved specific enzymatic systems to assist endophytes utilizing various nutrients or compounds from plants [13], which not only exerts assistance and protection to plants, but also stimulates production of biologically active substances in endophytes [14, 15]. Recent prospecting of microbial natural products revealed that the endophytic fungi or actinomycetes represent novel sources for discovery of secondary metabolites, because of their unique ecological environment and symbiotic relationship with host plants [15]. These advantages make endophytes very promising for efficient recycling of herb residues.

Huazhenghuisheng oral liquid (HOL) is a commercial compound TCM clinically applied for treatment of primary bronchial lung cancer and primary liver cancer. HOL is produced from 35 kinds of Chinese herbs, in which *Leonurus artemisia* (LA) is the major component accounted for almost 14% of the total weight. Taking HOL residues and LA residue as the objects, five endophytes were respectively evaluated in this study for their recycling capacities via solid-state fermentation, and the probiotic fungus *Aspergillus cristatus* CB10002 was screened out due to its well growth and the production of abundant metabolites. Subsequent identification and quantitative analysis focusing on medicinal valuable anthraquinones demonstrated the good potential of *A. cristatus* in the reclamation of herb residues. Further nutritional analysis also proposed a possibility to utilize the recycled HOL residues as a certain kind of compost, which may thoroughly solve the leftovers. Our studies provided a feasible approach for practical recycling of herb residues.

## Results

### Screening of endophytes for the potential recycling of herb residues

**Fig. 1:**
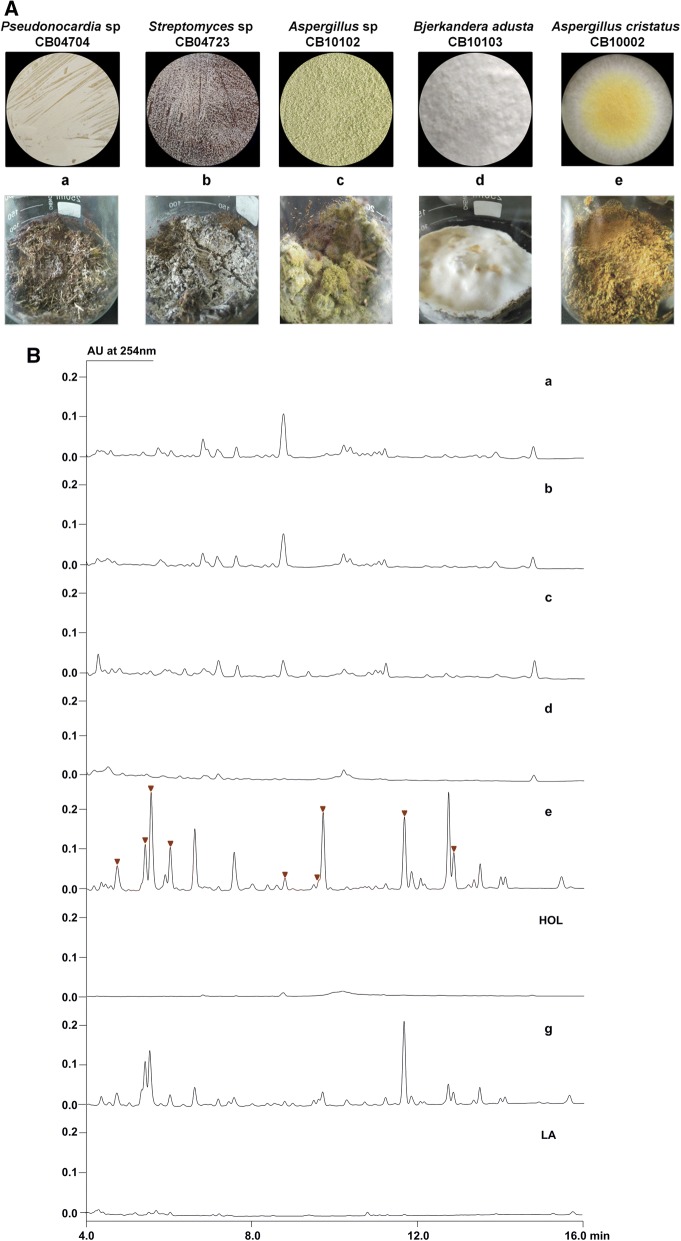
**A** The morphological appearances of selected endophytes either on sporulation plates or in solid state fermentation with HOL residues (a–e). **B** HPLC analysis of metabolites either from a-e (red marked peaks are anthraquinones), or from solid state fermentation of LA residue with CB10002 (g), using HOL residues (HOL) and LA residue (LA) as the control

Taking advantage of ecological features of endophytes, two endophytic actinomycetes and three endophytic fungi were selected from our microbial strain library to test their potential abilities for reutilization of HOL residues. These endophytes presented the different morphological appearances either on sporulation plates or in solid state fermentation, and endophytic fungi were clearly more suitable for growing on HOL residues (Fig. 1A). Comparing to the blank control, the HPLC analysis of crude extracts (Fig. 1B) indicated that all endophytes could use HOL residues to produce some metabolites, in which CB04704, CB04723 and CB10102 showed similar metabolic profiles with minor differences (Fig. 1B a–c), while CB10103 produced the least compounds (Fig. 1B d) in spite of its well growth. In contrast, CB10002 produced the most abundant metabolites (Fig. 1B e), in which a group of anthraquinones was very significant. Moreover, CB10002 could also use single LA residue to produce this group of compounds but with distinctly altered contents (Fig. 1B g), which may due to the varied nutritional components in different residues. Hence, CB10002 was selected for subsequent recycling studies in aspect of its excellent physiological characteristics and metabolic capacity.

### Structural characterization of anthraquinones and their possible biosynthetic relationships

**Fig. 2:**
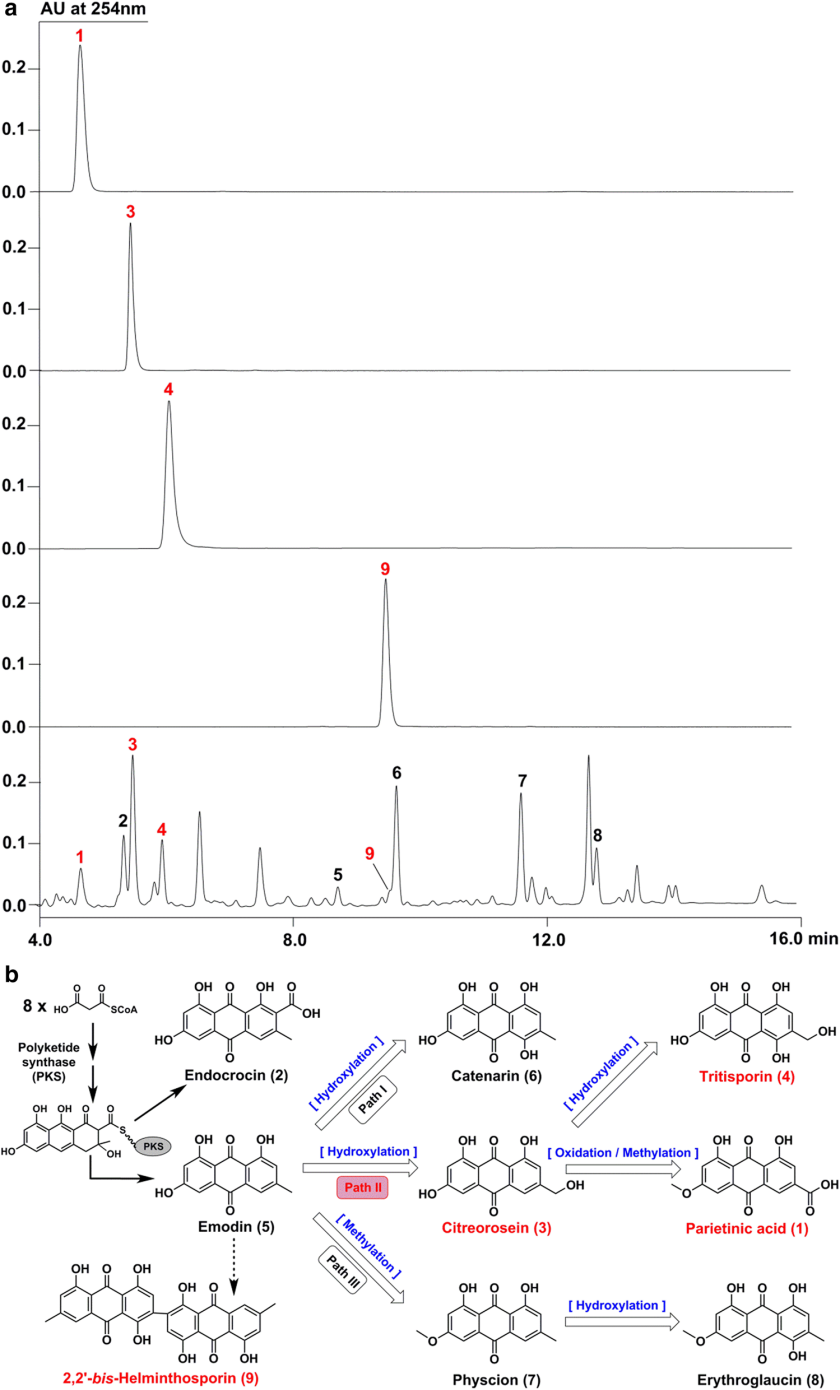
**a** Isolation and purification of nine anthraquinones (**1**–**9**) from the solid state fermentation of HOL residues with CB10002 (red marked compounds were first found in CB10002). **b** Structures of **1**–**9** and their deduced biosynthetic relationships with the common precursor **5** (the dashed arrow indicated uncertain steps)

Because anthraquinones constitute an important class of natural products with a wide range of medicinal activities, and also happen to be the main products detected from the solid state fermentation of HOL residues with CB10002, so we concentrated on the isolation, purification and structural elucidation of them (Fig. 2a). The obtained spectral data including UV, NMR and HRMS (Additional file 1) of target nine anthraquinones (**1**–**9**) were in good agreement with the published structures, and were respectively identified as parietinic acid (**1**), endocrocin (**2**), citreorosein (**3**), tritisporin (**4**), emodin (**5**), catenarin (**6**), physcion (**7**), erythroglaucin (**8**) and 2,2′-*bis*-helminthosporin (**9**) (Fig. 2b) [16–19]. **1**–**9** had been reported to originate from different species of lichen, plants and fungi [20, 21], and among them **4** was only isolated from *Helminthosporium tritici*-*vulgaris* [22], while **9** was newly discovered from the marine sponge-associated fungus *Talaromyces stipitatus* [18]. Based on our knowledge, **1**, **3**, **4** and **9** were first discovered in *A. cristatus*.

Although the scaffold of anthraquinones is known to be biosynthesized from malonyl-CoA by nonreduced polyketide synthase (PKS) in fungi [23], their real biosynthetic pathways especially about the post-PKS modifications have not been fully elucidated. Considering about the close related structures of **1**–**8**, recent progresses have revealed that **2** was probably the shunt product from the biosynthetic pathway of **5** [24], while **5** could be the primary precursor to generate other anthraquinones except **9** through three modification branch paths, in which the newly emerged **1**, **3** and **4** are likely derived from the same path II using **3** as the starting intermediate (Fig. 2b). In contrast, the generation of **9** or its monomer was still uncertain.

### Quantitative analysis of 1–9 in solid state fermentation of HOL residues

**Fig. 3:**
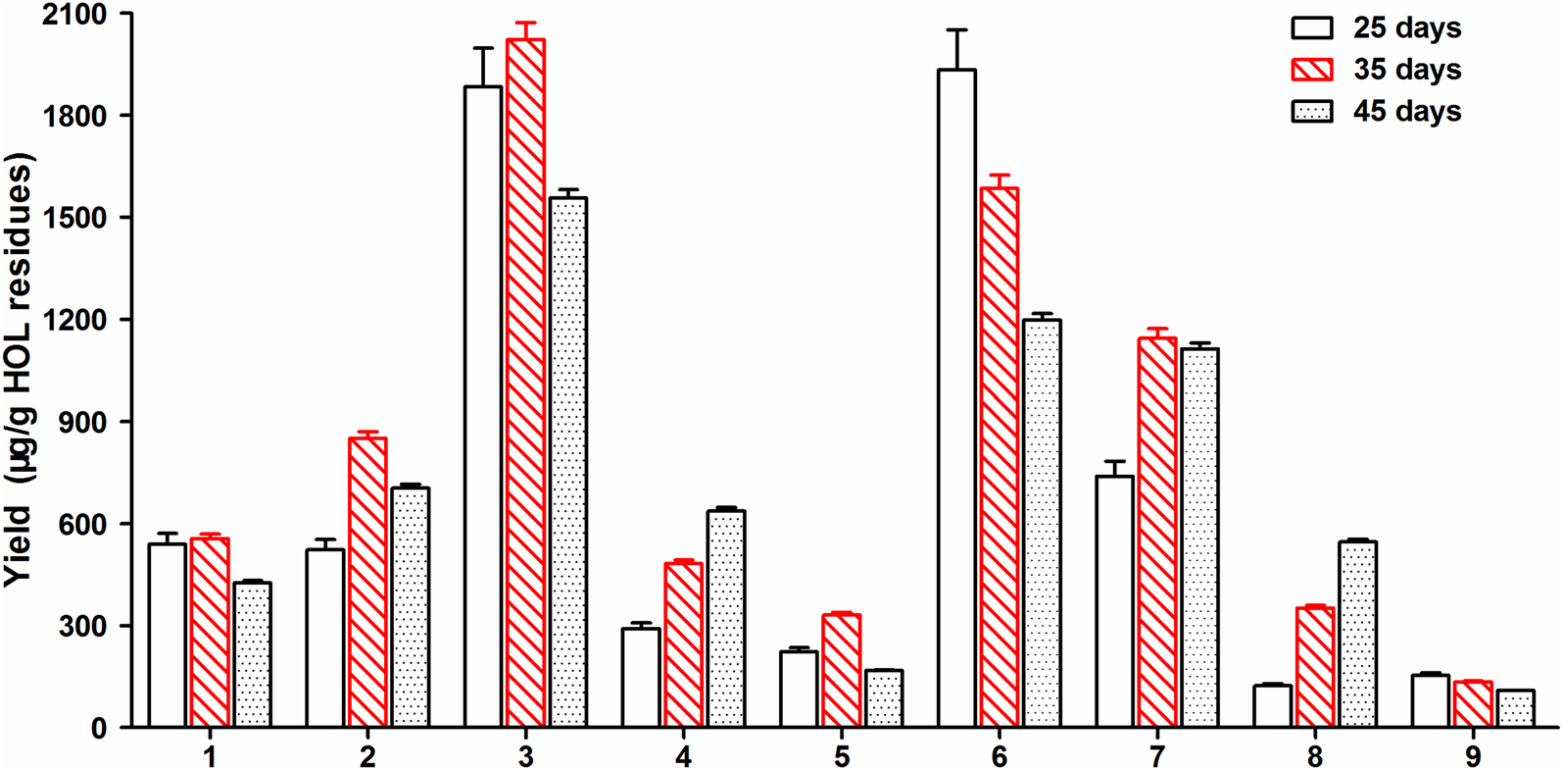
Quantitative analysis about the yields of **1**–**8** in solid state fermentation of HOL residues at different times

To determine the optimal time period for solid state fermentation and quantitatively evaluate the yields of **1**–**9**, samples from different fermentation times including 25, 35 and 45 days were analyzed (Fig. 3). The comparison indicated that the proper time for each compound to reach its maximum content were varied, but the total yield of **1**–**9** was peaked at 35 days for about 7.5 mg/g HOL residues, and the highest individual yield of about 2.0 mg/g HOL residues was obtained from **3**. In addition, the content changes of **4**, **6** and **8** were in different pattern with other compounds, and may be associated with the deduced biosynthetic routes (Fig. 2b). The increased contents of **4** and **8** at 45 days supported our hypothesis that they are respective downstream products of **3** and **7**, and therefore take longer time to be produced; while the contents of **3** and **6** were changed oppositely during 25 to 35 days, which hinted they may compete with each other in the hydroxylation paths (Fig. 2b). Taking **3** and **5** as the representatives, their yields from fermentation of HOL residues with CB10002 were very competitive comparing to those published data from either fermentation of fungi or extraction of plants (Table 1).

**Table 1:** Summary for the practical yields of 3 and 5 from different species and sources

Species	Sources	Yield of **3**	Yield of **5**	Refs.
*Talaromyces stipitatus* KUFA 0207	SSF of 15 kg rice	8.5 mg (0.6 mg/kg)	10.0 mg (0.7 mg/kg)	[18]
*Penicillium* sp. SCSGAF 0023	12 L liquid fermentation	7.0 mg (0.6 mg/L)	5.0 mg (0.4 mg/L)	[32]
*Chaetomium* sp. YMF 1.02105	SSF of 1 kg rice	5.0 mg (5.0 mg/kg)	8.0 mg (8.0 mg/kg)	[33]
*Microsphaeropsis* sp. 8875	SSF of 12 L agar	7.0 mg (0.6 mg/L)	15.0 mg (1.3 mg/L)	[34]
*Penicillium oxalicum* 2HL-M-6	75 L liquid fermentation	11.2 mg (0.1 mg/L)	14.7 mg (0.2 mg/L)	[35]
*Zopfiella longicaudata* IFM4630	SSF of 20 kg rice	1.5 mg (0.1 mg/kg)	NA	[36]
*Rumex aquaticus*	0.55 kg dried roots	3.4 mg (6.2 mg/kg)	5.2 mg (9.5 mg/kg)	[37]
*Reynoutria japonica*	10 kg dried roots	8.4 mg (0.8 mg/kg)	467.1 mg (46.7 mg/kg)	[38]
*Cassia nigricans*	0.5 kg dried leaf	32.4 mg (64.8 mg/kg)	32.0 mg (64.0 mg/kg)	[39]
*Aspergillus cristatus* CB10002	0.15 kg HOL residues	8.5 mg (56.7 mg/kg)	8.3 mg (55.3 mg/kg)	This study

*SSF* solid state fermentation, *NA* not available

### Nutritional analysis and comparison of herb residues under different conditions

**Fig. 4:**
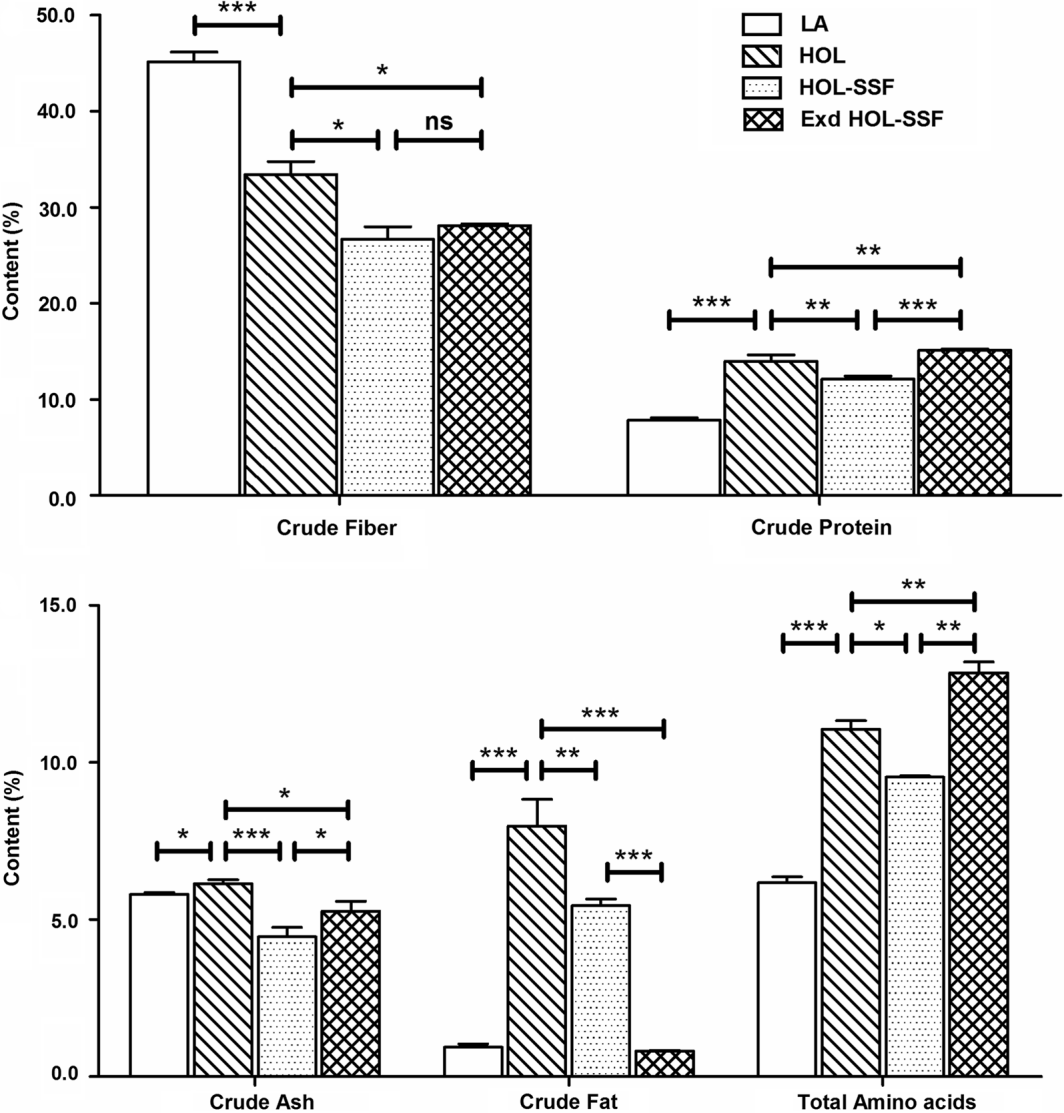
Comparison of nutrient contents in LA residue (LA) and HOL residues (HOL), as well as those in HOL residues after solid state fermentation (HOL-SSF) and subsequent extraction (Exd HOL-SSF) (*p < 0.05, **p < 0.01, ***p < 0.001)

By contrasting with those of HOL residues, the analysis of essential nutrient contents revealed that LA residue possessed 35.2% higher crude fiber, but about 44.0% lower crude protein and total amino acids, especially 88.3% much lower crude fat, whereas crude ash was similar (Fig. 4). Clearly, the compound HOL residues could provide more balanced and sufficient nutritional components. The nutritional comparison of HOL residues before and after 35-day solid state fermentation showed that the crude fat, crude protein and crude ash were actually more consumed than crude fiber by CB10002 (Fig. 4). After the extraction of fermented HOL residues, the contents of essential nutrients except crude fat were all increased at varied levels. The significantly improved levels of crude protein and total amino acids were even higher than those of original HOL residues, which probably attributed to the decomposition of CB10002 cells. On the contrary, the content of crude fat was remarkably further decreased 85.0%, because of its partial dissolution in methanol (Fig. 4). Overall, the nutritional composition of final leftovers after the microbial recycling of HOL residues presented distinct changes, which were somewhat similar to the compost process.

## Discussion

**Fig. 5:**
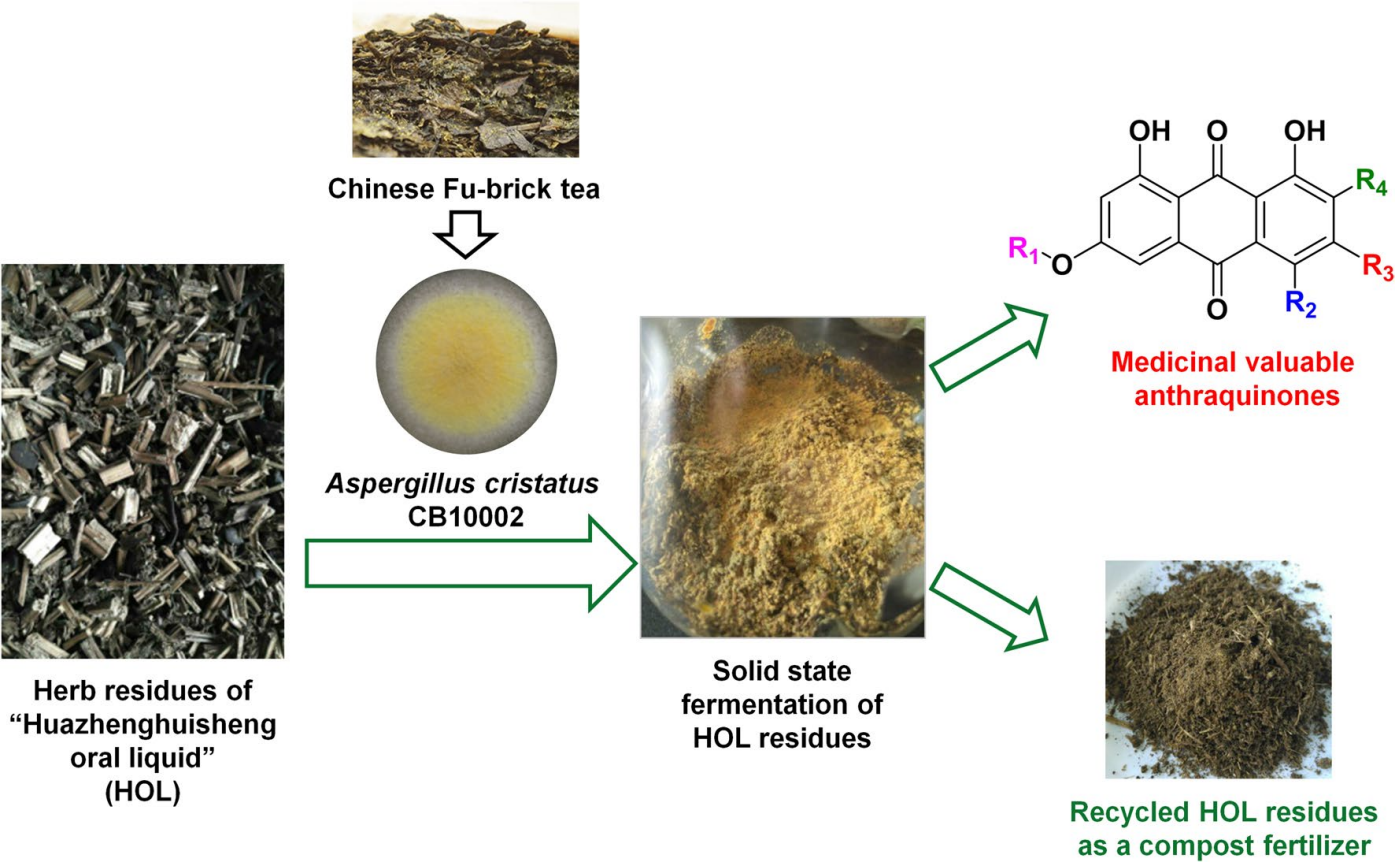
The sketch for practical recycling of HOL residues by *A. cristatus* CB10002

The prosperous development of CHM has generated massive herb residues, which become big issues about environmental pollution and waste of resources. The unique symbiotic relationship between endophytes and plants inspired us to explore the potential recycling of HOL residues by different endophytes. The evaluations suggested that *A. cristatus* CB10002, a probiotic fungus isolated from Chinese Fu-brick tea, was capable to reutilize either compound HOL residues or single LA residue for production of natural products, especially anthraquinones (Fig. 5).

As the dominant fungus involved in the fermentation of Fu-brick tea, *A. cristatus* has been reported to secret various extracellular enzymes like oxidase and cellulase for the utilization of tea stems and leafs, which exerts a major effect on organoleptic and healthy qualities of Fu-brick tea [25]. Previous studies have revealed that *A. cristatus* is able to produce various metabolites mainly including anthraquinones [19], benzaldehyde pigments [26] and diketopiperazines [27]. Besides, recent genomic investigation has also suggested *A. cristatus* as a probiotic fungus [28], because no known mycotoxin biosynthetic gene cluster was identified in its genome, which excluded the potential risk of toxic contamination. These merits of *A. cristatus* could be very beneficial for green and efficient recycling of herb residues.

Among identified nine anthraquinones derived from the recycling of HOL residues by CB10002, **1**–**8** have been reported to possess a number of biological activities including laxative, antioxidative, anti-inflammatory, antitumor and antimicrobial [29], some of them like **5** and **7** are also important medicinal ingredients occur in various CHM such as *Rheum palmatum*, *Polygonum cuspidatum*, and so on [30]. Remarkably, **3** has been claimed to show significant anti-obesity activity even better than resveratrol [18]. Subsequent quantitative analysis demonstrated that about 2.0 mg/g **3** and 7.5 mg/g total anthraquinones could be obtained after 35-day fermentation. Therefore, the simultaneous production of **1**–**9** with different origins during the fermentation of HOL residues indicated the good biosynthetic potential of *A. cristatus*, while the competitive yields of these medicinal valuable anthraquinones were economically beneficial for the recycling of HOL residues. On the other hand, the first discovery of **1**, **3**, **4** and **9** in *A. cristatus* also provided clues to thoroughly dissect the biosynthesis of emodin type anthraquinones. Various post-PKS modifications gave the abundant structural diversities of anthraquinones. As one of the key modification enzymes, cytochrome P450 monooxygenase (P450) has been generally recognized to catalyze site specific hydroxylation of anthraquinones, and also reported lately to be responsible for dimerization of anthraquinones [31]. Since the activated biosynthesis of **1**, **3**, **4** and **9** in CB10002 were all involved with P450 (Fig. 2b), this enzyme could be the main target regulated directly or indirectly by HOL residues, and critical for the production and content changes of **1**–**9**. The prospective genetic manipulation about the emodin pathway to address the biosynthesis of specific medicinal valuable anthraquinone such as **3**, shall further improve the applied potential of *A. cristatus* in reutilization of herb residues.

The nutritional analysis also revealed that the nutritional differences between single LA residue and compound HOL residues could contribute to the evident metabolic differences of CB10002 during the recycling process, in which the balanced and adequate nutrients of HOL residues could induce more comprehensive metabolic regulations in CB10002. Through the microbial fermentation, the nutrients of HOL residues were first converted by CB10002 for the increased microbial biomass and the production of natural products, and then the extracting process ameliorated the nutritional components of recycled HOL residues by reducing crude fat and increase of nitrogen sources, which proposed a possibility to directly use the final leftovers as a kind of compost organic fertilizer.

## Conclusions

In summary, the endophytic and probiotic fungus *A. cristatus* CB10002 isolated from Chinese Fu-brick tea was competent to reutilize HOL residues for the production of medicinal valuable anthraquinones, and four of them were first discovered in *A. cristatus*, whose biosynthesis may regulated by the induction of HOL residues. Besides, the promising yields of these anthraquinones were very competitive, which make the recycling of HOL residues economically beneficial. Further nutritional evaluations suggested that the microbial recycling process also exerted a certain composting effect on HOL residues, and hence proposed a potential resolution to dispose the final leftovers as a kind of compost organic fertilizer. Our studies demonstrated the competence of *A. cristatus* in microbial recycling, and provided a practical strategy for sustainable and value-added recycling of common herb residues.

## Methods

### General experimental procedures and data analyses

For analysis of nutrient ingredients in target herb residues, the contents of crude protein, crude fiber, crude fat and amino acids were tested by kieldahl apparatus B316 (Buchi, Fluwil, Switzerland), Scino FT350 fiber analyzer (Foss, Hilleroed, Denmark), Soxtec 2043 extraction system (Foss) and S433D amino acid analyzer (Sykam, Munich, Germany), respectively. For purification of compounds, column chromatography (CC) was carried out on silica gel (100–200 mesh and 300–400 mesh, domestic) or Sephadex LH-20 (GE, MA, USA), then semi-preparative reversed phase HPLC was performed on the Waters 1525 HPLC system equipped with a UV/Visible Detector and a Welch Ultimate AQ-C18 column (5 μm, 250 × 10 mm, Welch Materials Inc., Shanghai, China). For structural characterization of compounds, UV spectra, High-resolution mass spectra and NMR spectra were obtained on UV-1700 spectrometer (Shimadzu, Kyoto, Japan), LTQ Orbitrap ETD LC–MS system (Thermo Scientific, MA, USA) and 400 or 500 MHz NMR spectrometer (Brucker, Ettlingen, Germany), respectively. All experiments were performed in triplicate, and the data were expressed as an average value with standard deviation. Differences were considered statistically significant if p < 0.05.

### Microorganisms and cultivation conditions

All endophytes applied in this work were from the microbial strain library stored in Xiangya International Academy of Translational Medicine (XIATM), Central South University (Hunan, China). Among them, *Aspergillus cristatus* CB10002 was isolated from Fu-brick tea (Hunan, China), *Pseudonocardia* sp. CB04704 and *Streptomyces* sp CB04723 were isolated from *Tripterygium wilfordii* (Sichuan, China), *Aspergillus* sp CB10102 and *Bjerkandera adusta* CB10103 were isolated from *Sabia japonica* (Hunan, China). For sporulation on solid slants, CB10002 was grew on M40Y agar medium (10 g/L yeast extract, 20 g/L malt extract, 400 g/L sucrose and 20 g/L agar); CB04704 and CB04723 were grew on R2A medium (0.25 g/L trytone, 0.5 g/L casein acid hydrolysate, 0.5 g/L yeast extract, 0.25 g/L peptone, 0.5 g/L soluble starch, 0.5 g/L glucose, 0.3 g/L K_2_HPO_4_, 0.1 g/L MgSO_4_, 0.3 g/L sodium pyruvate); CB10102 and CB10103 were grew on potato dextrose agar (PDA) medium (200 g/L potato extract, 20 g/L glucose and 20 g/L agar); each strain was incubated at 30 °C for 7–10 days to collect mature spores. Similarly, M40Y liquid medium, tryptic soy broth (TSB) medium (17 g/L tryptone, 3 g/L soy peptone, 2.5 g/L K_2_HPO_4_, 5 g/L NaCl, 2.5 g/L glucose) and PDA liquid medium (without agar) were applied as seed medium to prepare inocula for CB10002, CB04704 and CB04723, CB10102 and CB10103, respectively. Generally, 100 μL of spore suspension was inoculated into a 250 mL Erlenmeyer flask containing 50 mL seed medium and incubated at 30 °C and 200 rpm for 2–3 days, then the seed broth was centrifuged and the mycelia were resuspended in 10 mL sterilized H_2_O.

### Solid-state fermentation of herb residues and HPLC analysis of metabolites

HOL residues were directly obtained from Hayaocihang Pharmaceutical Co. Ltd (Hunan, China), LA residue was prepared from *Leonurus artemisia* after aqueous extraction. All residues were dehydrated at 55 °C until constant weight and then ground into small fragments. Each 250 mL flask containing 10 g processed herb residues and 30 mL pure H_2_O was sterilized at 121 °C for 25 min, then inoculated with 10 mL inocula and well mixed; while the inoculation of 10 mL pure H_2_O was applied as the blank reference. For comparison of different endophytes, fermentation flasks were incubated at 30 °C for 35 days, while for time course evaluation of CB10002, various fermentation times including 25, 35 and 45 days were applied. After fermentation, the entire solid mixture in each 250 mL flask was extracted with a total of 300 mL methanol for three times (100 mL and 60 min per time) under interval stirring. The combined extracts were evaporated under vacuum and redissolved in 20 mL methanol. Then 50 μL of concentrate was diluted to 1 mL and filtered through a 0.22 μm polytetrafluoroethylene (PTFE) needle filter, and 10 μL of sample was analyzed on the Waters E2695 HPLC system equipped with a photo-diode array (PDA) detector and a Welch AQ-C18 column (5 μm, 250 × 4.6 mm). The mobile phase consisted of buffer A (ultrapure H_2_O containing 0.1% HCO_2_H) and buffer B (chromatographic grade CH_3_CN containing 0.1% HCO_2_H) at a flow rate of 1 mL/min. A gradient program (50% buffer A to 5% buffer A from 0 min to 10 min; 5% buffer A from 10 min to 15 min; 5% buffer A to 50% buffer A from 15 min to 18 min; 50% buffer A from 18 min to 20 min) was applied.

### Isolation, purification and quantitative analysis of anthraquinones

About 150.0 g solid products derived from the fermentation of HOL residues with CB10002 were collected, and extracted three times with 4 L methanol. The concentrated 7.9 g crude extract was subjected to silica gel CC by sequentially using petroleum ether (PE)/ethyl acetate (EAC) and EAC/methanol (MT) gradient elution systems to obtain fractions A-D, in which the concentration of polar solvent was stepwisely increased by 10%. Fraction D was further isolated on silica gel CC by using EAC/MT gradient elution system to give three subfractions, which were purified by semi-preparative HPLC (using the same solvent system and gradient elution program in HPLC analysis, at a flow rate of 3.0 mL/min) to obtain pure compounds **1** (1.9 mg), **2** (8.0 mg), **3** (8.5 mg) and **4** (6.2 mg), respectively. Fractions B and C were further isolated on silica gel CC by using PE/EAC gradient elution system to give four subfractions, which were purified by semi-preparative HPLC to obtain pure compounds **5** (8.3 mg), **6** (18.9 mg), **7** (11.2 mg) and **8** (10.3 mg), respectively. To isolate the minor compound **9**, a total of 830.0 g solid products were similarly processed to give 0.5 g fraction A, which was subjected to Sephadex LH-20 CC by using isocratic elution of MT to obtain the crude extract, and then purified by semi-preparative HPLC to finally afford pure compound **9** (32.0 mg). The yield of a certain emodin-type anthraquinone was quantified based on the calibration curve of emodin standard (98.6% pure, National Institute of Metrology, China).

## Additional file

**Additional file 1.** Supplementary data.

## Data Availability

Not applicable.
