# Supplementary material for: Recycling of Chinese herb residues by endophytic and probiotic fungus Aspergillus cristatus CB10002 for the production of medicinal valuable anthraquinones
Source: Microb Cell Fact. 2019 Jun 4;18:102. doi: 10.1186/s12934-019-1150-9 (PMC6547571; doi:10.1186/s12934-019-1150-9)
Supplement: Supplementary file 1 — Additional file 1. Supplementary data. [file 12934_2019_1150_MOESM1_ESM.docx]

**Supplementary Data**

**Recycling of Chinese herb residues by endophytic and probiotic fungus *Aspergillus cristatus* CB10002 for the production of medicinal valuable anthraquinones**

Wenping Kong ^1^, Chengshuang Huang ^1^, Jie Shi ^1^, Yu Li ^1^, Xinxin Jiang ^2^, Quwen Duan ^2^, Yong Huang ^1, 4^, Yanwen Duan^*1, 3, 4^, and Xiangcheng Zhu ^*1, 3, 4^

^1^Xiangya International Academy of Translational Medicine, Central South University,

^2^Hayaocihang Pharmaceutical Co. Ltd,

^3^Hunan Engineering Research Center of Combinatorial Biosynthesis and Natural Product Drug Discovery, and

^4^National Engineering Research Center of Combinatorial Biosynthesis for Drug Discovery, Changsha, Hunan, 410013, China;

^*^To whom correspondents should be addressed: Dr. Xiangcheng Zhu, Central South University, Tongzipo Road，#172，Yuelu District，Changsha, Hunan 410013，China. Tel: (86) 731 8265 0539; Fax: (86) 731 8265 0551; Email: seanzhu1996@aliyun.com or Prof. Yanwen Duan, Email: ywduan66@sina.com.

**Spectra data for structural characterization of isolated anthraquinones 1-9**

Compound **1**: yellow powder; UV (CH_3_OH) λmax 225.4, 285.8, 435.6; HRESI-MS, m/z 315.0498 (M­­+H)^+^; 1H-NMR (DMSO-*d_6_*, 500 MHz) : δ =13.40 (1H, s),8.08 (1H, s), 7.66 (1H, s), 7.19 (1H, s),6.82 (1H, s), 3.90 (3H, s); 13C-NMR (DMSO-*d_6_*, 125 MHz) : 185.8, 182.5, 165.0, 163.8, 161.5, 157.9, 136.9, 131.9, 129.6, 123.9, 118.6, 117.6, 111.8, 108.0, 105.1, 56.2.

Compound **2**: yellow powder; UV (CH_3_OH) λmax 221.9, 266.8, 288.2, 440.5; HRESI-MS, m/z 313.0359 (M­­−H)^−^; 1H-NMR (CD_3_OD-*d_4_*, 400 MHz) : δ =7.60 (1H, s), 7.19 (1H, d, *J*=2.0 Hz), 6.57 (1H, d, *J*=2.4 Hz), 2.45 (3H, s); 13C-NMR (DMSO-*d_6_*, 125 MHz) : 191.7, 182.7, 167.4, 166.7, 165.0, 159.9, 145.3, 136.8, 134.3, 130.9, 122.0, 115.3, 110.6, 110.3, 109.1, 20.3.

Compound **3**: yellow powder; UV (CH_3_OH) λmax 224.2, 266.8, 288.2, 441.7; HRESI-MS, m/z 287.0553 (M­­+H)^+^; 1H-NMR (DMSO-*d_6_*, 500 MHz) : δ =12.17 (1H, s), 7.59 (1H, s), 7.20 (1H, s), 7.05 (1H, s), 6.48 (1H, s), 4.57 (3H, s); 13C-NMR (DMSO-*d_6_*, 125 MHz) : 189.0, 181.5, 166.9, 164.7, 161.5, 152.4, 135.0, 132.9, 120.8, 116.9, 114.1, 109.6, 108.4, 108.0, 62.1.

Compound **4**; orange-red powder; UV (CH_3_OH) λmax 230.1, 254.9, 278.7, 487.8; HRESI-MS, m/z 325.0316 (M­­+Na)^+^; 1H-NMR (DMSO-*d_6_*, 500 MHz): δ =7.29 (1H, s), 7.02 (1H, s), 6.38 (1H, s), 4.58 (2H, s); 13C-NMR (DMSO-*d_6_*, 125 MHz): 187.0, 186.2, 164.9, 163.6, 156.1, 154.6, 143.0, 134.6, 125.0, 111.8, 110.7, 108.3, 107.3, 57.4.

Compound **5**: yellow powder; UV (CH_3_OH) λmax 221.9, 266.8, 288.2, 439.3; HRESI-MS, m/z 271.0602 (M­­+H)^+^; 1H-NMR ((CD_3_)_2_CO)-*d_6_*, 400 MHz): δ =12.19 (1H, s), 12.08 (1H, s), 7.55 (1H, s), 7.25 (1H, s), 7.13 (1H, s), 6.66 (1H, s), 2.47 (3H, s); 13C-NMR ((CD_3_)_2_CO)-*d_6_*, 100 MHz): 191.6, 182.2, 166.6, 166.3,163.36, 149.5, 136.6, 134.2, 124.9, 121.5, 114.5, 110.3, 109.8, 108.9, 22.3.

Compound **6**: orange-red powder; UV (CH_3_OH) λmax 230.1, 256.1, 277.5, 303.7, 490.3; HRESI-MS, m/z 287.0553 (M+H)^+^; 1H-NMR (DMSO-*d_6_*, 400 MHz): δ =13.14 (1H, s), 12.13 (1H, s), 7.21 (1H, s), 7.10 (1H, s), 6.55 (1H, s), 2.23 (3H, s); 13C-NMR (DMSO-*d_6_*, 100 MHz): 186.8, 186.6, 164.7, 163.6, 156.7, 155.9, 139.3, 134.6, 129.1, 111.7, 111.6, 110.3, 109.6, 108.2, 15.8.

Compound **7**: yellow powder; UV (CH_3_OH) λmax 223.0, 266.8, 287.0, 435.6; HRESI-MS, m/z 285.0758 (M+H)^+^; 1H-NMR (DMSO-*d_6_*, 500 MHz): δ =12.19 (1H, s), 11.98 (1H, s), 7.54 (1H, s), 7.21 (1H, d, *J* = 2.5 Hz), 6.89 (1H, d, *J* = 2.5 Hz), 3.94 (3H, s),2.43 (3H, s); 13C-NMR (DMSO-*d_6_*, 125 MHz): 190.0, 181.3, 166.2, 164.4, 161.5, 148.6, 134.9, 132.9, 124.3, 120.6, 113.5, 109.9, 107.7, 106.7, 56.4, 21.6.

Compound **8**: orange-red powder; UV (CH_3_OH) λmax 231.3, 256.1, 276.3, 304.9, 490.3; HRESI-MS, m/z 301.0107 (M+H)^+^; 1H-NMR (CDCl_3_-*d*, 500 MHz): δ =13.38 (1H, s), 12.47 (1H, s), 12.39 (1H, s), 7.41 (1H, s), 7.14 (1H, s), 6.70 (1H, s), 3.95 (3H, s),2.36 (3H, s); 13C-NMR (CDCl_3_-*d*, 125 MHz): 189.0, 186.6, 166.5, 165.3, 158.0, 157.4, 140.8, 135.3, 129.4, 125.2, 112.0, 110.8, 107.6, 107.1, 56.2, 16.7.

Compound **9**: red powder; UV (CH_3_OH) λmax 225.4, 256.1, 294.1, 468.4, 530.5; HRESI-MS, m/z 539.0965 (M+H)^+^; 1H-NMR (DMSO-*d_6_,* 500 MHz): δ =12.83 (1H, s), 12.06 (1H, s), 7.19 (1H, s), 7.13 (1H, s), 6.76 (1H, s), 2.28 (3H, s); 13C-NMR (DMSO-*d_6_*, 125 MHz): 189.3, 181.3, 164.5, 161.1, 148.1, 133.0, 131.6, 123.6, 120.6, 113.1, 109.0, 108.7, 107.7, 21.4.

**Figure S1. The calibration curve of emodin standard**
